# Precision Diagnosis in *APOL1* Kidney Disease With the p.N264K M1 Protective Variant

**DOI:** 10.1001/jamanetworkopen.2026.1452

**Published:** 2026-03-11

**Authors:** Elena Martinelli, Juntao Ke, Atlas Khan, Janewit Wongboonsin, David R. Vanderwall, Tze Y. Lim, Dominick Santoriello, Yask Gupta, Michelle T. McNulty, Satoshi Koyama, Sidhant Puntambekar, Andrew S. Bomback, Pietro Canetta, Matthias Kretzler, Giovanni Montini, William Morello, Umberto Maggiore, Enrico Fiaccadori, Loreto Gesualdo, Gian Marco Ghiggeri, Eduardo Araújo Oliveira, Ana Cristina Simoes e Silva, Pavan K. Bendapudi, Joshua Motelow, Christine K. Garcia, Dirk S. Paul, Slavé Petrovski, David B. Goldstein, David J. Friedman, Jai Radhakrishnan, Fangming Lin, Sumit Mohan, Gerald B. Appel, Moin A. Saleem, Pradeep Natarajan, Friedhelm Hildebrandt, Rik Westland, Vivette D. D’Agati, Rasheed Gbadegesin, Ali G. Gharavi, Martin R. Pollak, Krzysztof Kiryluk, Matthew G. Sampson, Simone Sanna-Cherchi

**Affiliations:** 1 Division of Nephrology, Department of Medicine, Columbia University Irving Medical Center, New York, New York; 2 Division of Pediatric Nephrology, Boston Children’s Hospital, Boston, Massachusetts; 3 Renal Division, Faculty of Medicine, Siriraj Hospital, Mahidol University, Bangkok, Thailand; 4 Division of Nephrology, Brigham and Women’s Hospital, Boston, Massachusetts; 5 Harvard Medical School, Boston, Massachusetts; 6 Bumrungrad Genomic Medicine Institute and Department of Medicine, Bumrungrad International Hospital, Bangkok, Thailand; 7 Department of Cell Biology, Harvard Medical School, Boston, Massachusetts; 8 Unit of Genomic Variability and Complex Diseases, Department of Medical Sciences, University of Turin, Turin, Italy; 9 The Renal Pathology Laboratory of the Department of Pathology and Cell Biology, Columbia University, New York, New York; 10 Lübeck Institute of Experimental Dermatology, University of Lübeck, Lübeck, Germany; 11 Program in Medical and Population Genetics, Broad Institute of Harvard and the Massachusetts Institute of Technology, Cambridge; 12 Cardiovascular Research Center and Center for Genomic Medicine, Massachusetts General Hospital, Boston; 13 Division of Nephrology, Department of Internal Medicine, University of Michigan, Ann Arbor; 14 Pediatric Nephrology, Dialysis and Transplant Unit, Fondazione IRCCS Ca’ Granda Ospedale Maggiore Policlinico, Milan, Italy; 15 UO Nefrologia, Dipartimento di Medicina e Chirurgia, Università di Parma, Parma, Italy; 16 Department of Precision and Regenerative Medicine and Ionian Area (DiMePre-J) Nephrology, Dialysis and Transplantation Unit, University of Bari Aldo Moro, Bari, Italy; 17 Nephrology, Dialysis, and Transplantation, IRCCS Istituto Giannina Gaslini, Genoa, Italy; 18 Unidade de Nefrologia Pediátrica, Departamento de Pediatria, Laboratório Interdisciplinar de Investigação Médica, Faculdade de Medicina, Universidade Federal de Minas Gerais (UFMG), Belo Horizonte, MG, Brazil; 19 Division of Hematology and Blood Transfusion Service, Massachusetts General Hospital, Boston,; 20 Division of Hemostasis and Thrombosis, Beth Israel Deaconess Medical Center, Boston, Massachusetts; 21 Division of Critical Care and Hospital Medicine, Department of Pediatrics, Columbia University Medical Center, New York, New York; 22 Medicine/Pulmonary, Allergy, and Critical Care Medicine, Columbia University Irving Medical Center, New York, New York; 23 Precision Medicine Initiative and the Center for Precision Medicine and Genomics, Columbia University, New York, New York; 24 Centre for Genomics Research, Discovery Sciences, BioPharmaceuticals R&D, AstraZeneca, Cambridge, United Kingdom; 25 Actio Biosciences Inc, San Diego, California; 26 Division of Nephrology, Department of Medicine, Beth Israel Deaconess Medical Center, Boston, Massachusetts; 27 Division of Pediatric Nephrology, Department of Pediatrics, Columbia University, New York, New York; 28 Bristol Renal, University of Bristol, and Bristol Royal Children’s Hospital, Bristol, United Kingdom; 29 Department of Pediatric Nephrology, Amsterdam UMC – Emma Children’s Hospital, location University of Amsterdam, Amsterdam, the Netherlands; 30 Division of Nephrology, Department of Pediatrics, Duke University School of Medicine, Durham, North Carolina; 31 Department of Pediatrics and Medicine, Harvard Medical School, Boston, Massachusetts

## Abstract

**Importance:**

The *APOL1* M1 (p.N264K) variant protects against G2-associated *APOL1* focal segmental glomerulosclerosis (FSGS) and chronic kidney disease (CKD). However, the utility of knowing an individual’s M1 status in guiding kidney disease diagnosis and other clinical scenarios remains underexplored.

**Objective:**

To test 2 hypotheses: (1) in patients with *APOL1* high-risk (HR) genotype kidney disease with at least 1 G2 allele, M1 can distinguish *APOL1* CKD from non-*APOL1* CKD; (2) in people with *APOL1* low-risk (LR) genotypes, M1 is independently associated with protection against FSGS and CKD.

**Design, Setting, and Participants:**

Retrospective case-control study using data from 2 tertiary care hospitals (Columbia University Irving Medical Center and Mass General Brigham Biobank) and population-based data (the UK Biobank [UKB], Electronic Medical Records and Genomics [eMERGE-III], and All of Us [AoU]). Participants were individuals with a diagnosis of FSGS or steroid-resistant nephrotic syndrome (SRNS), individuals with CKD, and controls.

**Exposures:**

Exposures included the M1 variant (p.N264K) obtained from exome or genome sequencing data, sex, and genetic ancestry.

**Main outcome and measure:**

The main outcome was the presence or absence of kidney disease, defined as FSGS or non-FSGS CKD, compared with non–kidney disease controls. Association between the M1 variant and disease status was assessed using odds ratios (ORs).

**Results:**

A total of 107 696 individuals (54 994 [51.1%] female; 8779 [8.2%] with African ancestry, 78 475 [72.9%] with European ancestry, and 16 129 [15.0%] with multiethnic ancestry), including 3460 with FSGS or SRNS, 24 382 with non-FSGS CKD kidney disease, and 79 854 controls were enrolled in the discovery cohort. In the *APOL1*-HR group (1413 participants), M1 was significantly inversely associated with FSGS or SRNS cases compared with controls without kidney disease (OR, 0.20; 95% CI, 0.04-0.63; *P* = 3.69 × 10^−3^). Among individuals with CKD with *APOL1*-HR genotypes, M1 was 4 times more frequent in those whose CKD was not due to FSGS or SRNS. Importantly, electronic health record and biopsy review identified an alternative, non-*APOL1* cause for CKD in nearly all *APOL1*-HR-M1 cases. There was no association between individuals with *APOL1*-LR genotypes with M1 and protection against CKD or FSGS.

**Conclusions and relevance:**

In this case-control study of 107 696 individuals, presence of an *APOL1*-HR genotype M1 was significantly associated with protection against kidney disease, suggesting that it may have a role as a genetic modifier. Patients with CKD with an A*POL1*-HR genotype and M1 should be evaluated for an alternative and potentially treatable cause of their CKD.

## Key Points

**Question:**

What is the association of the protective *APOL1* M1 (p.N264K) variant with kidney disease risk in individuals with *APOL1* high-risk (*APOL1*-HR) and low-risk (*APOL1*-LR) genotypes?

**Findings:**

In this case-control study of 107 696 individuals, the M1 variant was associated with improved diagnostic precision for kidney disease causes among individuals with *APOL1*-HR genotypes and showed no independent association with protection in the general population of individuals with *APOL1*-LR genotypes.

**Meaning:**

These findings suggest that presence of the M1 variant in individuals with *APOL1*-HR genotypes may point to a distinct and potentially actionable mechanism of kidney disease.

## Teaser

This case-control study evaluates whether, in patients with *APOL1* high-risk genotype kidney disease with at least 1 G2 allele, M1 can distinguish *APOL1* chronic kidney disease from non-*APOL1* chronic kidney disease.

## Introduction

Two West African ancestry–associated variants in the apolipoprotein L1 (*APOL1*) gene, G1 (rs73885319 and rs60910145) and G2 (rs71785313), contribute to the 5-times higher incidence of chronic kidney disease (CKD) among Black Americans compared with European Americans, who preferentially carry the G0 wild-type (WT) haplotype.^1^

A spectrum of nondiabetic kidney diseases is associated with *APOL1* high-risk (HR) genotypes, defined as G1G1, G1G2, and G2G2. Among these conditions, *APOL1*-HR genotypes confer large odds ratios (ORs) for focal segmental glomerulosclerosis (FSGS), a pattern of glomerular injury underlying a large fraction of idiopathic nephrotic syndrome, when compared with individuals with *APOL1* low-risk genotypes (*APOL1*-LR), G0G0, G0G1, G0G2.^2,3,4^

However, only 5% to 8% of individuals with *APOL1-*HR genotypes actually develop FSGS, suggesting that environmental and/or genetic factors modify their penetrance.^5^ For genetic testing to be informative in both ruling in or out a diagnosis and, hence, providing enhanced and equitable care, the genotype of interest must display a high deterministic value on the clinical presentation of the tested individual. This is especially true in genetics-first or genetics-early settings, when genetic testing is administered early in the diagnostic workup and its results might influence physicians’ decision-making regarding further diagnostic testing (eg, imaging studies and kidney biopsy) or treatment (eg, avoidance of immunosuppression and starting targeted therapies). *APOL1*-HR genotypes, despite their large effect size for risk of FSGS (ORs up to 30)^2^ and CKD (ORs up to 4),^6^ have high prevalence but low penetrance in individuals of genetic African ancestry, consequently complicating the diagnostic, prognostic, and therapeutic workup for *APOL1* kidney disease. On one hand, early knowledge of *APOL1* risk variant status during the diagnostic workup can improve diagnostic precision, avoid unnecessary treatments, and identify individuals who might benefit from specific *APOL1* targeting approaches.^7,8^ On the other hand, *APOL1*-HR genotypes are present in approximately 13% of self-reported Black US residents^9,10^ and in up to 50% of individuals living in West Africa^11,12,13^; this high frequency, together with their incomplete penetrance and limited predictive value, poses risks for so-called label diagnosis that might result in undesired effects, such as incomplete diagnostic work-up (eg, avoidance of kidney biopsy) or modifying standard-of-care management decisions with the unwanted possibility of increasing disparities.

We, among others, reported on the protective association between the *APOL1* p.N264K missense variant (M1; rs73885316) and *APOL1* kidney disease and, specifically, FSGS.^14,15^ The *APOL1* M1, when coinherited on the same haplotype with the high-risk G2 *APOL1* allele,^16^ virtually negates this association, effectively rendering G2-containing *APOL1* HR genotypes comparable with nonrisk genotypes.^14,15,17,18^

These new insights support the potential importance of genotyping the *APOL1* M1 variant in routine genetic testing.^19,20,21^ In the setting of presymptomatic screening or transplant donor evaluation, the presence of M1 in individuals with *APOL1* G1G2 and G2G2 genotypes—traditionally considered to be at high risk—would lower the estimated risk of them developing CKD and FSGS. In the setting of patients with already diagnosed steroid-resistant nephrotic syndrome (SRNS) or FSGS, CKD, or end-stage kidney disease, finding *APOL1* G1G2 or G2G2 and M1 on genetic testing would strongly suggest that they did not have *APOL1* kidney disease. Rather, these patients would require a different and complete diagnostic workup to identify immune, toxic, structural, or other causes for their disease and enact adequate therapies. These observations led to our first hypothesis: in individuals with *APOL1*-HR genotypes, M1 genotyping will improve the diagnostic precision for *APOL1* kidney disease and will help distinguishing it from non-*APOL1* CKD or FSGS.

Additionally, in the original study by Hung et al,^15^ the authors observed a suggestive protective association of M1 even in individuals with *APOL1*-LR genotypes. If confirmed, this protective association of M1 in *APOL1*-LR genotype carriers would imply a potentially deleterious effect of *APOL1* even in individuals with G0, which has not been previously demonstrated. It would additionally suggest an independent absolute protective role of M1 against CKD development. Current understanding of *APOL1*-associated kidney toxicity contradicts a toxic role of G0.^22,23,24^ Therefore, our study aimed to investigate a second hypothesis: in individuals with *APOL1*-LR genotypes, M1 may have a protective association against FSGS and CKD.

To test these hypotheses, we studied 107 696 individuals from 2 large tertiary hospitals’ biobanks at Columbia University Irving Medical Center (CUIMC) and the Massachusetts General Brigham Health system. We then validated the results in 23 955 individuals of African ancestry from the Electronic Medical Records (EMR) and Genomics (eMERGE-III) project, the UK Biobank (UKB), and the All of Us (AoU) research program.

## Methods

For this case-control study, written informed consent was collected from all participating individuals and/or their guardians in accordance with the Columbia University institutional review board and the policy on bioethics and human biologic samples of AstraZeneca. Results conform to Strengthening the Reporting of Observational Studies in Epidemiology (STROBE) reporting guideline. Additional details on study methods are available in the eMethods in Supplement 1.

### Cohorts and DNA Sequencing

The total discovery cohort included 54 304 individuals from CUIMC^25^ and 53 392 individuals from the Mass General Brigham Biobank^26^ with genome or exome sequencing (eTable 1 in Supplement 1). This dataset included 27 842 individuals with CKD and 79 854 individuals not meeting the CKD criteria who were defined as controls. Among individuals with CKD, 3460 had biopsy-confirmed FSGS or SRNS and 24 382 had CKD not attributable to FSGS or SRNS (here defined as non-FSGS CKD). A total of 79 854 controls were identified from individuals without evidence of CKD. KING version 2.3.1 (KING)^27^ was used to remove related individuals; principal components were calculated using PLINK2^28^ to infer genetic ancestries according to the 1000 Genome populations,^29^ and *APOL1* G1, G2, and M1 genotypes were extracted from sequencing data (eFigure 1 in Supplement 1).

### Replication in Public Biobanks

We replicated our findings in individuals of African ancestry across 3 biobanks: the EMR and eMERGE-III project,^30,31^ the UKB,^32^ and the AoU research program.^33,34,35^ Ancestry was defined using a random forest-based machine learning approach. We trained and tested the random forest model using subjects from the 1000 Genomes Project with known ancestry labels.^29^

To define individuals with CKD and controls in these 3 biobanks, we applied our validated CKD e-phenotyping algorithm.^31^ To classify individuals with CKD caused by FSGS or SRNS, we developed a selection pipeline based on relevant *International Classification of Diseases, Ninth Revision (ICD-9)* codes (eTable 2 and eFigure 2 in Supplement 1), generating 3 cohorts aligned with the discovery study: FSGS or SRNS, non-FSGS CKD, and controls.

### Statistical Analyses

In the discovery cohorts, pairwise comparisons of M1 variant prevalence across phenotypic categories (FSGS or SRNS, non-FSGS CKD, and controls) were performed using Fisher exact test and regression analyses were performed using the Firth bias-reduced logistic regression, which applies a penalized likelihood approach to mitigate small-sample bias and issues of separation.^36,37^ Covariates included sex and genetic ancestry in the full-cohort models. Conversely, models restricted to individuals of African ancestry were adjusted for sex only. In the external biobank datasets, M1 variant prevalence was compared across phenotypic groups using the Cochran-Mantel-Haenszel test, with biobank and sex as stratifying variables. Statistical significance was defined as a 2-sided *P* value less than .05. All analyses and forest plots were conducted with R version 4.5.1 (R Project for Statistical Computing) and the bar plots were generated using GraphPad Prism version 10.6.1 for Windows (GraphPad Software).

## Results

### Baseline Characteristics of the Discovery Cohort and *APOL1* Variants

The combined cohort included 107 696 individuals (54 994 [51.1%] female; 8779 [8.2%] African ancestry, 78 475 [72.9%] European ancestry, and 16 129 [15.0%] multiethnic ancestry), including 3460 with FSGS, 24 382 with non-FSGS CKD, and 79 854 controls (Table 1). Among those with FSGS, 1510 of 3460 were female (43.6%); sex distributions were similar in non-FSGS CKD (9961 of 24 382 [40.8%]), while controls had a slightly higher proportion of female participants (43 523 of 79 854 [54.5%]).

**Table 1.** Baseline Characteristics of the Study Population^a^

Characteristic	FSGS or SRNS (n = 3460)	Non-FSGS CKD (n = 24 382)	Control (n = 79 854)
Sex			
Female	1510 (44.0)	9961 (41.0)	43 523 (55.0)
Male	1950 (56.0)	14 421 (59.0)	36 270 (45.0)
Unknown	0	0	61 (<0.1)
Ancestry			
African	511 (14.8)	1975 (8.1)	6293 (7.9)
East Asian	71 (2.1)	558 (2.3)	1571 (2.0)
European	1759 (50.8)	17 775 (72.9)	58 941 (73.8)
Multiethnic	971 (28.1)	3464 (14.2)	11 694 (14.6)
South Asian	147 (4.2)	575 (2.4)	1068 (1.3)
Unknown	1 (<0.1)	35 (0.1)	287 (0.4)
*APOL1* Risk			
Low	3140 (91.0)	23 940 (98.0)	79 203 (99.0)
G0G0	2948 (85.0)	22 982 (94.0)	75 778 (95.0)
G0G1	120 (3.5)	574 (2.4)	1965 (2.5)
G0G2	72 (2.1)	384 (1.6)	1460 (1.8)
High	320 (9.2)	442 (1.8)	651 (0.8)
G1G1	158 (4.6)	191 (0.8)	248 (0.3)
G1G2	125 (3.6)	196 (0.8)	306 (0.4)
G2G2	37 (1.1)	55 (0.2)	97 (0.1)
*APOL1* p.N264K			
Wild-type	3442 (99.0)	24 278 (100)	79 493 (100)
Heterozygous	18 (0.5)	103 (0.4)	352 (0.4)
Homozygous	0	1 (<0.1)	9 (<0.1)

Abbreviations: CKD, chronic kidney disease; FSGS, focal segmental glomerulosclerosis; SRNS, steroid-resistant nephrotic syndrome.

^a^ Demographic, ancestry, and *APOL1* genotype distributions are shown for individuals with FSGS, CKD, and controls. Genetic ancestry was imputed from principal components analysis derived from the 1000 Genome populations reference panel. *APOL1* risk status is classified as low-risk (G0G0, G0G1, or G0G2) or high-risk (G1G1, G1G2, or G2G2).

Genetic ancestry distributions reflected the diversity of the dataset. Of 3460 individuals with FSGS, 511 (14.8%) were of African ancestry, 71 (2.1%) of East Asian ancestry, 1759 (50.8%) of European ancestry, 971 (28.1%) of multiethnic ancestry, and 147 (4.2%) of South Asian ancestry. Of 24 382 individuals in the non-FSGS CKD group, 1975 (8.1%) were of African ancestry, 558 (2.3%) were of East Asian ancestry, 17 775 (72.9%) were of European ancestry, 3464 (14.2%) were of multiethnic ancestry, and 575 (2.4%) were of South Asian ancestry. Of 79 854 in the control group, 6293 (7.9%) were of African ancestry, 1671 (2.0%) were of East Asian ancestry, 58 941 (73.8%) were of European ancestry, 11 694 (14.6%) were of multiethnic ancestry, and 1068 (1.3%) were of South Asian ancestry.

*APOL1* risk genotypes followed expected patterns across disease groups. *APOL1*-HR genotypes were observed in 320 of 3460 individuals (9.2%) of all-ancestries FSGS (273 of 320 [85.3%] of African ancestry, 46 of 320 [14.4%] of multiethnic ancestry, and 1 [0.3%] of European ancestry), 442 of 24 382 individuals (1.8%) with CKD, (400 of 442 [90.5%] of African ancestry, 41 of 442 [9.3%] of multiethnic ancestry, and 1 of 442 [0.2%] of East Asian ancestry), and 651 of 19 854 (0.8%) controls (613 of 651 [94.2%] of African ancestry, 34 of 651 [5.2%] of multiethnic ancestry and 4 of 651 [0.6%] of unknown ancestry), with the remainder in each group classified as *APOL1*-LR.

As expected, individuals of African ancestry had the highest allelic frequencies for G1 and G2 as compared with all the other groups (Table 2). *APOL1* M1 allelic frequency and distribution among groups are summarized in eTable 3 in Supplement 1. A total of 61 controls did not have sex data (all *APOL1*-LR), and for 287 controls (4 *APOL1*-HR), 35 non-FSGS CKD (all *APOL1*-LR) cases, and 1 FSGS (*APOL1*-LR) case, a genetic ancestry category could not be estimated; these 384 cases were removed from the Firth bias-reduced logistic regression.

**Table 2.** Distribution of *APOL1* Allelic and Genotype Frequencies Across Genetic Ancestries and Clinical Phenotypes^a^

Alleles and genotypes	African	East Asian	European	Multiethnic	South Asian	FSGS	CKD	Controls
Alleles								
G0	11 502 (65.5)	4396 (99.9)	3572 (99.8)	31 017 (96.2)	3572 (99.8)	6088 (88.0)	46 922 (96.2)	154 981 (97.0)
G1	3758 (21.4)	4 (0.1)	41 (<0.1)	657 (2.0)	5 (0.1)	561 (8.1)	1152 (2.4)	2767 (1.7)
G2	2298 (13.1)	0	25 (<0.1)	584 (1.8)	3 (0.1)	271 (3.9)	690 (1.4)	1960 (1.2)
Genotypes								
G0G0	4009 (45.7)	2197 (99.9)	78 410 (99.9)	15 009 (93.1)	1782 (99.6)	2948 (85.2)	22 982 (94.3)	75 778 (94.9)
G0G1	2092 (23.8)	2 (0.1)	39 (0.1)	509 (3.2)	5 (0.3)	120 (3.5)	574 (2.4)	1965 (2.5)
G0G2	1392 (15.9)	0	25 (<0.1)	490 (3.0)	3 (0.2)	72 (2.1)	384 (1.6)	1460 (1.8)
G1G1	547 (6.2)	1 (0.1)	1 (<0.1)	48 (0.3)	0	158 (4.6)	191 (0.8)	248 (0.3)
G1G2	572 (6.5)	0	0	52 (0.3)	0	125 (3.6)	196 (0.5)	306 (0.4)
G2G2	167 (1.9)	0	0	21 (0.1)	0	37 (1.1)	55 (0.2)	97 (0.1)

Abbreviations: CKD, chronic kidney disease; FSGS, focal segmental glomerulosclerosis.

^a^ G0 is the reference allele; G1 and G2 are risk variants associated with kidney disease. Genotype frequencies reflect all possible combinations of these alleles. The *APOL1* G1 and G2 risk alleles are largely restricted to individuals of African ancestry, with minimal representation in non-African populations. High-risk genotypes (G1G1, G1G2, and G2G2), are enriched among individuals with FSGS compared with those with CKD or unaffected controls.

### *APOL1* M1 Variant Association With FSGS or SRNS

**Figure 1.**
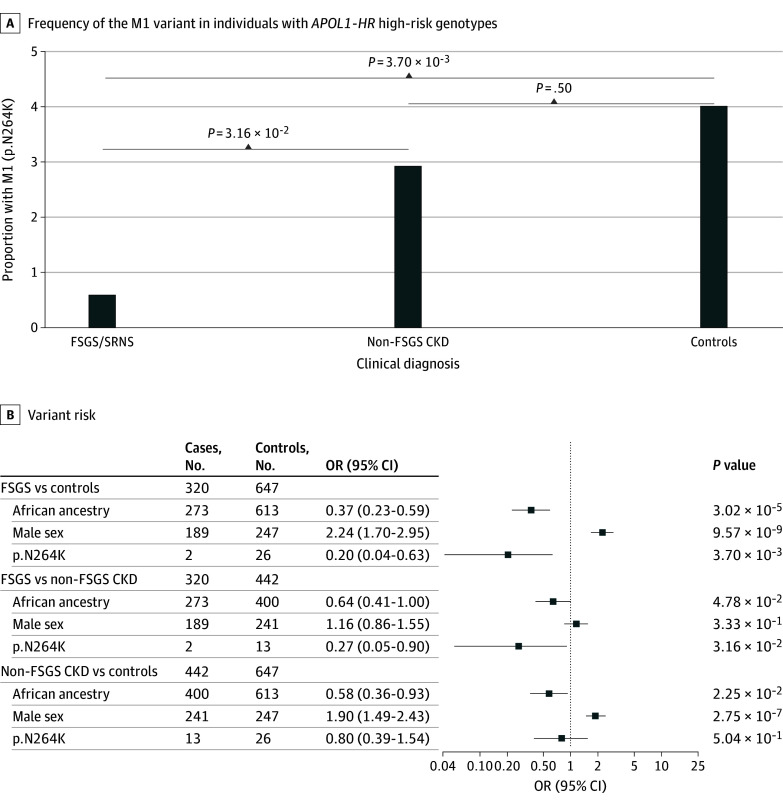
M1 Association With Focal Segmental Glomerulosclerosis (FSGS) or Steroid-Resistant Nephrotic Syndrome (SRNS), Non-FSGS Chronic Kidney Disease (CKD), and Controls in *APOL1* High-Risk (HR) Genotypes The bar plots show the frequency of the M1 variant (p.N264K) among individuals with *APOL1*-HR genotypes, stratified by clinical diagnosis: FSGS or SRNS, non-FSGS CKD, and unaffected controls. Forest plots show odds ratios (ORs) and 95% CIs from the Firth bias-reduced logistic regression analysis comparing individuals with FSGS or SRNS with controls and with non-FSGS CKD, as well as non-FSGS CKD with controls. Covariates included genetic ancestry (African and non-African; non-African as reference), sex (female as reference) and M1 (wild-type as reference). LR indicates low-risk; OR, odds ratio.

To investigate our first hypothesis (ie, that in individuals with *APOL1*-HR, M1 genotyping will improve the precision of diagnosing *APOL1* kidney disease and distinguishing it from non-*APOL1* kidney disease), we investigated the association of the M1 variant in the *APOL1*-HR group (G1G1, G1G2, and G2G2) with CKD cases and controls. M1 was nominally inversely associated with CKD status overall and when compared with controls (15 of 762 patients [1.97%] vs 26 of 651 [3.99%]; Fisher exact OR, 0.48; 95% CI, 0.24-0.96; *P* = 2.62 × 10^−2^; Firth regression OR, 0.54; 95% CI, 0.28-1.02; *P* = 5.83 × 10^−2^). This inverse association between M1 and cases was primarily associated with the FSGS or SRNS subgroup (2 of 320 patients [0.63%]) compared with controls (26 of 651 patients [3.99%]; Fisher exact OR, 0.15; 95% CI, 0.02-0.61; *P* = 1.86 × 10^−3^; 26 of 647 patients; Firth regression OR, 0.20; 95% CI, 0.04-0.63; *P* = 3.70 × 10^−3^) (Figure 1; eTable 4 in Supplement 1). However, M1 was not significantly associated with non-FSGS CKD cases (13 of 442 patients [2.94%]) or controls (Fisher exact OR, 0.73; 95% CI, 0.34-1.49; *P* = .41; 13 of 442 patients; Firth regression OR, 0.80, 95% CI, 0.39-1.54; *P* = .50) (Figure 1; eTable 4 in Supplement 1). Furthermore, harboring M1 was approximately 4 times more likely to be associated with non-FSGS CKD than FSGS or SRNS cases (13 of 442 patients [2.94%] vs 2 of 320 [0.63%], Fisher exact OR, 4.81; 95% CI, 1.08-44.21; *P* = 3.15 × 10^−2^; Firth regression OR, 3.70; 95% CI, 1.11-18.96; *P* = 3.16 × 10^−2^) (Figure 1; eTable 4 in Supplement 1).

Restricting these analyses to individuals with *APOL1* G2-containing HR genotypes (G1G2 and G2G2) supported these findings (eFigure 3 in Supplement 1). Moreover, since G1, G2, and M1 are significantly more frequent in individuals of African continental genetic ancestry, we conducted sensitivity analyses in the 673 CKD cases and 613 controls of genetic African ancestry only. This showed nearly identical results as in the whole dataset of individuals with *APOL1*-HR genotypes (eTable 5 in Supplement 1).

We next proceeded to validate these results in 3 large biobanks. Both individually in eMERGE-III, UKB, and AoU, as well as in aggregate, these analyses showed a direction-consistent effect size between M1 and FSGS or SRNS cases as compared with controls and individuals with non-FSGS CKD, although the results were not statistically significant, possibly due to the limited number of FSGS or SRNS cases in these population-based biobanks (Cochran-Mantel-Haenszel stratified by Biobank and sex, FSGS vs controls common OR, 0; 95% CI, 0.00-1.26; *P* = .11; FSGS vs non-FSGS CKD common OR, 0; 95% CI, 0.00-1.73; *P* = .26) (eTable 6, eFigure 4 in Supplement 1).

Altogether, these data confirm that in individuals with *APOL1*-HR genotypes, the presence of M1 is associated with nearly complete protection against *APOL1* FSGS or SRNS. Furthermore, in non-FSGS CKD cases with *APOL1*-HR genotypes, the presence of M1 suggests a non-*APOL1* cause and that an alternative, potentially treatable cause of their disease should be pursued.

### Electronic Health Record (EHR) Review and Non-*APOL1* Causes for CKD in *APOL1*-HR M1 Carriers

The large-scale human genetics studies reported previously indicate that the presence of M1 protects against *APOL1* kidney disease, especially FSGS or SRNS. Consequently, individuals with CKD who have *APOL1*-HR and the M1 protective missense variant should have an alternative cause for their CKD.

To investigate this, we conducted a detailed retrospective review of the EHR for all *APOL1*-HR cases with CKD who harbored the M1 variant in the discovery cohort. A detailed description of the findings is available in eAppendix, eFigure 5, and eTable 12 in Supplement 1. In summary, in the FSGS or SRNS group, the 2 *APOL1*-HR-M1 cases (0.63%) had presentations that suggest that the FSGS or SRNS phenotype in these 2 cases is associated with non-*APOL1* mechanisms, and may simply represent the background prevalence of FSGS or SRNS in cases with *APOL1*-HR-M1; in the non-FSGS CKD group with *APOL1*-HR genotypes and M1, we were able to identify an alternative, non-*APOL1* mediated cause of CKD in 10 of 13 individuals (76.9%). Importantly, in the 3 cases in whom we could not identify an alternative cause of CKD, the diagnostic workup was incomplete.

### Protective Association of M1 Independent of *APOL1*

**Figure 2.**
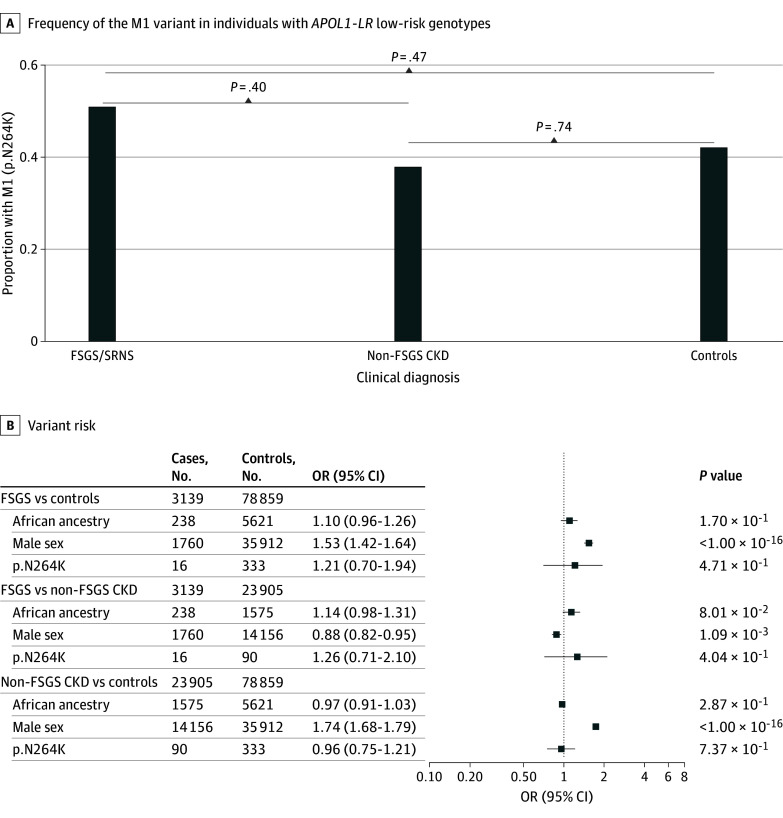
M1 Association With Focal Segmental Glomerulosclerosis or Steroid-Resistant Nephrotic Syndrome (FSGS or SRNS), Non-FSGS Chronic Kidney Disease (CKD), and Controls in *APOL1* Low-Risk (LR) Genotypes The bar plots show the frequency of the M1 variant (p.N264K) among individuals with *APOL1* LR genotypes, stratified by clinical diagnosis: FSGS or SRNS, non-FSGS CKD, and unaffected controls. Forest plots show ORs and 95% CIs from the Firth bias-reduced logistic regression analysis comparing individuals with FSGS or SRNS with controls and with non-FSGS CKD, as well as non-FSGS CKD with controls. Covariates included genetic ancestry (African and non-African; non-African as reference), sex (female as reference) and M1 (wild-type as reference). HR indicates high risk; OR, odds ratio.

We next formally tested the hypothesis that M1 might protect against CKD even in those without an *APOL1* HR genotype. In individuals with *APOL1*-LR genotypes (G0G0, G0G1, and G0G2), the prevalence of M1 was similar and not statistically different across groups (Figure 2; eTables 7-8 in Supplement 1). These data suggested no protective association of M1, independent of *APOL1*-HR genotypes, for FSGS or SRNS and CKD. The results were not different when restricting the analysis to individuals with *APOL1*-LR genotypes of genetic African ancestry alone (eTable 9 in Supplement 1).

Nevertheless, there is mounting evidence of a potential heterozygous risk for G1 and G2 that might have confounded these analyses.^11^ Therefore, we conducted a subset analysis restricted to only individuals with G0G0 genotypes across all ancestries. Here, we also found no evidence of an independent protective association of M1 (eTable 10 in Supplement 1).

We again proceeded to validate these results in 3 large biobanks. Both individually in eMERGE-III, UKB, and AoU, as well as in aggregate, these analyses showed no support for a protective association of M1, independent of *APOL1*-HR genotypes (eTable 11, eFigure 4 in Supplement 1).

## Discussion

The identification of the *APOL1* M1 (p.N264K) modifier variant in a patient treated in Ghana for Trypanosoma infection in 2013,^18^ followed by the studies in 2019 and 2023,^15,38^ marked an important phase in the efforts devoted to identifying genetic modifiers of *APOL1* kidney disease. Now that M1 has been discovered and validated, the next phase of this effort is understanding the extent and magnitude in which the knowledge of a person’s M1 status can improve the precision of our care for them to provide better prognosis and risk stratification, as well as to identify individuals who will benefit the most from drugs directly and indirectly targeting *APOL1*.^7,8^

In this case-control study, we investigated the association of the M1 variant across different *APOL1* kidney risk genotypes with distinct phenotypic groups within our large-scale, well-characterized study cohort followed by validation in 3 external biobanks. Specifically, we first investigated whether M1 could enhance diagnostic precision for people with *APOL1*-HR genotypes (prevalently of African ancestry and multiethnic genetic ancestries) who are diagnosed with kidney disease. Second, we tested if M1 could operate as an independent protective modifier against kidney disease in the general *APOL1*-LR population (ie, in individuals across non-African and African ancestries) in absence of *APOL1*-HR genotypes.

Therefore, we first assessed if M1 would help in correctly diagnosing *APOL1* kidney disease by identifying individuals who, despite harboring recessive *APOL1*-HR genotypes, had their CKD attributable to non-*APOL1* causes, which would require specific diagnostic workup and might be amenable to targeted therapies. As expected, M1 was significantly depleted in FSGS or SRNS *APOL1*-HR cases compared with non-CKD controls. Interestingly, *APOL1*-HR cases with non-FSGS CKD only had a marginally lower frequency of M1 as compared with non-CKD controls, but this difference was not statistically significant, and they were approximately 4-fold more likely to harbor M1 as compared with FSGS or SRNS cases. These data suggested that a fraction of *APOL1*-HR individuals with non-FSGS CKD and who carried the *APOL1* M1 variant did not have *APOL1* kidney disease but another cause of CKD that would require specific diagnostic workup and treatment. In fact, detailed retrospective EHR review identified a likely alternative non-*APOL1* cause of disease for 2 of 2 FSGS and 10 of 13 non-FSGS CKD cases that were *APOL1*-HR-M1. In particular, the cases spanned across different causes of CKD with a clear independent cause, including diabetic nephropathy, amyloidosis, systemic lupus erythematosus, obstructive uropathy, IgA nephropathy, ANCA-associated vasculitis, immunocomplex mesangial proliferative and sclerosing glomerulonephritis, and steroid-sensitive nephrotic syndrome. Kidney biopsy slides were available for in-depth review in 2 of 13 cases, and both of them had an alternative cause of glomerular disease not attributable to *APOL1*. Only 2 cases had a clinical diagnosis of hypertension-mediated kidney disease, of which 1 had nonnephrotic range proteinuria that both might be attributable to *APOL1*, although the possibility of a label diagnosis without a complete diagnostic workup is possible.

Interestingly, even in the FSGS or SRNS group, the 2 cases that were found to harbor a *APOL1* high-risk genotype together with the M1 variant had an unlikely *APOL1*-mediated cause for FSGS, as they were either diagnosed with congenital nephrotic syndrome or had pediatric onset (aged 8 years), nonprogressive FSGS (eAppendix in Supplement 1). In fact, *APOL1*-HR genotypes are known to increase risk for kidney disease in young adults, but to a lesser extent in children.^39^ Previous studies including pediatric populations showed that the median age of disease onset was older in children who were *APOL1*-HR compared with children who were *APOL1*-LR (median [IQR], 11.5 [9.5-12.5] vs 4.5 [1.5-12.5] years), whereas the occurrence of congenital nephrotic syndrome in *APOL1*-HR individuals remains anecdotical and possibly coincidental.^40^ These data, if further validated, suggest that M1 completely protects against *APOL1* FSGS or SRNS, such that in individuals with *APOL1*-HR-M1 genotypes and FSGS or SRNS, the latter is likely a coincidental diagnosis in the context of primary or secondary podocytopathy which would require a further diagnostic pursuit (ie, testing for Mendelian disease or antinephrin antibodies). Similarly, in individuals with non-FSGS CKD, the presence of M1 suggests an alternative, non-*APOL1*, cause of kidney disease that would require complete serologic, histologic and radiologic diagnostic workup. While this recommendation is particularly important in the presence of M1, physicians should nevertheless apply standard-of-care diagnostics, including kidney biopsy, to all individuals with *APOL1*-HR, as M1 does not explain the full incomplete penetrance of *APOL1* risk variants, and many of these individuals have alternative etiologies for their CKD. Now that a new *ICD-10 *code, N07.B, has been introduced for *APOL1* kidney disease, this work should help in correctly assigning it to patients. Implementation should be easy and cost-effective since most commercial and noncommercial testing is based on gene panels, therefore the M1 variant is already captured and only needs to be reported.^41,42^ Moreover, in a kidney transplant setting, the knowledge of M1 would allow reclassification of *APOL1*-HR-M1 donors as nonrisk, thus expanding the donors pool. Similarly, this knowledge would improve risk assessment for kidney outcome of recipients of *APOL1*-HR-M1 kidneys. In asymptomatic individuals, detection of the M1 variant via genetic screening may indicate the absence of elevated susceptibility to *APOL1*-associated nephropathy and facilitate more precise risk stratification in population-level studies. A second goal of our study was to formally test the possible independent protective role of M1 in absence of *APOL1*-HR genotypes. Hence, we evaluated the difference in M1 prevalence among individuals with *APOL1*-LR genotypes across the same phenotypic categories and did not detect a protective effect of M1 for FSGS or SRNS nor for non-FSGS CKD as compared with control groups. This finding is consistent with the role of the M1 variant as a genetic modifier that exclusively neutralizes the deleterious effects of G2, without independently preventing non-*APOL1* kidney disease. The fact that M1 did not impact individuals with a G0 allele is consistent with the well-established observation that G0 is the ancestral, nonrisk allele not associated with increased susceptibility to kidney disease. Furthermore, it suggests that *APOL1*-targeted therapies to prevent or treat kidney disease would not work for individuals who are only carrying LR G0- or G1-containing genotypes.

### Limitations

This study has some limitations. First, the discovery cohorts include 8% individuals of African ancestry and 15% individuals of AMR ancestry. These numbers, while reflective of the prevalence of the genetic ancestries in the US population, might have negatively affected power, although our results reached statistical significance due to the enrichment of individuals with FSGS or SRNS and CKD in our discovery datasets. Second, our discovery cohort lacked granularity in age of onset and additional unmeasured clinical factors that could have influenced the presence of FSGS or CKD; nonetheless, our analysis focused on hard end points and relied on robust genotypic and phenotypic classifications that minimize the impact of missing clinical details. We also analyzed 3 large biobanks (eMERGE-III, AoU, and UKB) for validation. Our findings revealed a consistent direction of association with lower odds of having a diagnosis of FSGS or SRNS when carrying the M1 variant in individuals with *APOL1*-HR genotypes, though this difference did not reach statistical significance. These biobanks in fact include a much healthier population less enriched for kidney disease than our discovery dataset, especially for FSGS or SRNS. Nevertheless, it is notable that all individuals with *APOL1*-HR-M1 within the non-FSGS CKD group had *ICD-9* codes that indicate clinical diagnoses unrelated to *APOL1* kidney disease, such as diabetes, sarcoidosis, or systemic lupus erythematosus.

## Conclusions

The findings of this case-control study suggest that *APOL1* genotyping is incomplete without testing for M1. In fact, routine incorporation of M1 in the *APOL1* genetic testing might enable physicians and genetic counselors to reclassify patients with an *APOL1*-HR genotype as not having kidney disease due to *APOL1* and prompt the investigation for an alternative and potentially treatable cause of CKD not associated with *APOL1*. This will not only help improve diagnosis, prognosis, and treatment strategies, but may also avoid the possibility of substandard treatment for vulnerable populations, where ancestry and the presence of *APOL1*-HR genotypes might result in an unsubstantiated label diagnosis of *APOL1* kidney disease with resulting incomplete diagnostic workup and inadequate care. Importantly, across biobanks in the US and Europe and across genetic ancestries, we confirmed the role of M1 as a genetic modifier only of the G2 allele, reinforcing the evidence that *APOL1* G0 genotypes are not inherently deleterious. This study paves the way for increasingly personalized approaches to kidney disease diagnosis, management, and treatment, especially for individuals of African ancestry.

## Notes

Supplement 1. eMethods. eAppendix. EHR Review Suggests Non-*APOL1* Causes for CKD in *APOL1*-HR M1 Carriers eFigure 1. Design of the Study eFigure 2. Case Selection Workflow for Biobank Replication Analyses eFigure 3. Prevalence of M1 in *APOL1* G2 Containing HR Genotypes eFigure 4. Prevalence of M1 Across Biobanks and *APOL1* Genotypes eFigure 5. Kidney Biopsies Review of Case #3 and Case #9 eTable 1. Exome Capture Kits Used for Whole Exome Sequencing in the CUIMC Cohort eTable 2. *ICD-9* Codes eTable 3. Allelic Frequency M1 Across Ancestries and Phenotypes eTable 4. Inverse Association of M1 With FSGS or SRNS *APOL1*-HR Genotypes, in Both Adjusted and Unadjusted Models eTable5. Concordant M1 Prevalence in Individuals With *APOL1*-HR With Genetic African Ancestry eTable 6. Biobank-Based Insights Into M1 Prevalence Across *APOL1*-HR Genotypes eTable 7. M1 Prevalence in *APOL1*-LR eTable 8. Absence of Association of M1 With FGSS/SRNS or Non-FSGS CKD in *APOL1*-LR Genotypes eTable 9. M1 Prevalence in Individuals With *APOL1*-LR of Genetic African Ancestry eTable 10. *APOL1* G0G0 Test for M1 eTable 11. Biobank-Based Insights Into M1 Prevalence Across *APOL1*-LR Genotypes eTable 12. Clinical Characteristics of Individuals With *APOL1*-HR Genotypes Carrying the M1 Protective Variant eReferences.

Supplement 2. eTable. *ICD-9* Codes

Supplement 3. Nonauthor Collaborators

Supplement 4. Data Sharing Statement
