# Supplementary material for: Precision Diagnosis in APOL1 Kidney Disease With the p.N264K M1 Protective Variant
Source: JAMA Netw Open. 2026 Mar 11;9(3):e261452. doi: 10.1001/jamanetworkopen.2026.1452 (PMC12980251; doi:10.1001/jamanetworkopen.2026.1452)
Supplement: Supplement 1. — eMethods. eAppendix. EHR Review Suggests Non-APOL1 Causes for CKD in APOL1-HR M1 Carriers eFigure 1. Design of the Study eFigure 2. Case Selection Workflow for Biobank Replication Analyses eFigure 3. Prevalence of M1 in APOL1 G2 Containing HR Genotypes eFigure 4. Prevalence of M1 Across Biobanks and APOL1 Genotypes eFigure 5. Kidney Biopsies Review of Case #3 and Case #9 eTable 1. Exome Capture Kits Used for Whole Exome Sequencing in the CUIMC Cohort eTable 2. ICD-9 Codes eTable 3. Allelic Frequency M1 Across Ancestries and Phenotypes eTable 4. Inverse Association of M1 With FSGS or SRNS APOL1-HR Genotypes, in Both Adjusted and Unadjusted Models eTable5. Concordant M1 Prevalence in Individuals With APOL1-HR With Genetic African Ancestry eTable 6. Biobank-Based Insights Into M1 Prevalence Across APOL1-HR Genotypes eTable 7. M1 Prevalence in APOL1-LR eTable 8. Absence of Association of M1 With FGSS/SRNS or Non-FSGS CKD in APOL1-LR Genotypes eTable 9. M1 Prevalence in Individuals With APOL1-LR of Genetic African Ancestry eTable 10. APOL1 G0G0 Test for M1 eTable 11. Biobank-Based Insights Into M1 Prevalence Across APOL1-LR Genotypes eTable 12. Clinical Characteristics of Individuals With APOL1-HR Genotypes Carrying the M1 Protective Variant eReferences. [file jamanetwopen-e261452-s001.pdf]

Supplemental Online Content

Martinelli E, Ke J, Khan A, et al. Precision diagnosis in APOL1 kidney disease with the p.N264K M1 protective variant. *JAMA Netw Open*. 2026;9(3):e261452. doi:10.1001/jamanetworkopen.2026.1452

eMethods.

eAppendix. EHR Review Suggests Non-*APOL1* Causes for CKD in *APOL1*-HR M1 Carriers

eFigure 1. Design of the Study

eFigure 2. Case Selection Workflow for Biobank Replication Analyses

eFigure 3. Prevalence of M1 in APOL1 G2 Containing HR Genotypes

eFigure 4. Prevalence of M1 Across Biobanks and *APOL1* Genotypes

eFigure 5. Kidney Biopsies Review of Case #3 and Case #9

eTable 1. Exome Capture Kits Used for Whole Exome Sequencing in the CUIMC Cohort

eTable 2. *ICD9* Codes

eTable 3. Allelic Frequency M1 Across Ancestries and Phenotypes

eTable 4. Inverse Association of M1 With FSGS/SRNS *APOL1*-HR Genotypes, in Both Adjusted and Unadjusted Models

eTable5. Concordant M1 Prevalence in *APOL1*-HR Individuals With Genetic African Ancestry

eTable 6. Biobank-Based Insights Into M1 Prevalence Across *APOL1*-HR Genotypes

eTable 7. M1 Prevalence in *APOL1*-LR

eTable 8. Absence of Association of M1 With FGSS/SRNS or Non-FSGS CKD in APOL1-LR Genotypes

eTable 9. M1 Prevalence in *APOL1*-LR Individuals of Genetic African Ancestry

eTable 10. *APOL1* G0G0 Test for M1

eTable 11. Biobank-Based Insights Into M1 Prevalence Across *APOL1*-LR Genotypes

eTable 12. Clinical Characteristics of Individuals With *APOL1*-HR Genotypes Carrying the M1 Protective Variant

eReferences.

This supplemental material has been provided by the authors to give readers additional information about their work.

© 2026 Martinelli E et al. *JAMA Network Open*.

eMethods

Written informed consent was collected from all participating individuals seen at Columbia University Irving Medical Center (and collaborating Institutions) and/or their guardians in accordance with the Columbia University Institutional Review Board (Protocol AAAC7385) and the policy on bioethics and human biologic samples of AstraZeneca. All internationally recruited patients and/or their guardians were consented according to the Declaration of Helsinki and in compliance with the local ethic committees, as part of the parent IRB protocol approved at Columbia University.

Cohorts and DNA sequencing

The total discovery cohort included 107,696 patients with detailed clinical data and with genome or exome sequencing (WGS, WES). This cohort was comprised of 54,304 individuals from Columbia University Irving Medical Center(CUIMC)[1] and by 53,392 individuals from the Mass General Brigham Biobank (MGBB)[2]. The CUIMC biobank was composed of 12,051 individuals with WGS and 42,080 with WES (**Table S1**); all individuals of the MGBB had WES data.

Overall, the combined dataset included 27,842 individuals with CKD, defined by the following criteria alone or in combination: eGFR < 90 mL/min/1.73 m^2, persistent urinary abnormalities, imaging findings of structural abnormalities of kidney and the urinary tract, or presence of any ICD10 codes for CKD (MGBB only). 79,854 individuals not meeting the CKD criteria were defined as controls. Among cases with CKD, we further discriminated between those with biopsy confirmed FSGS or steroid resistant nephrotic syndrome (SRNS) (N=3,460) and CKD cases without FSGS/SRNS (N=24,382). Controls from CUIMC were defined as either individuals with disorders unrelated to kidney disease with normal kidney function, or individuals enrolled as controls or healthy family members to diverse studies (N= 34,926), as previously described[3-5]. Controls from MGBB were selected from individuals without any ICD10 code for CKD (N=44,928).

All analyses were done on unrelated samples after removing individuals with genetic relatedness up to two degrees using KING v2.3.0[6]. Principal components (PCs) were calculated using PLINK2 based on the linkage disequilibrium (LD)-pruned single nucleotide polymorphisms (SNPs)[7]. Then, genetic ancestries were inferred according to the 1000 Genome populations. We extracted G1 and G2 genotypes from sequencing data to delineate *APOL1*-HR and LR genotypes, and additionally extracted the M1 variant genotypes in these groups (e**Figure 1**).

© 2026 Martinelli E et al. *JAMA Network Open*.

Replication in public biobanks

We replicated our findings in individuals of African Ancestry across three biobanks: the Electronic Medical Records (EMR) and Genomics (eMERGE-III; N=2,381) project[8,9], the UK BioBank (UKBB, N=4,440)[10], and the All of Us (AoU, N=17,134) research program[11]. Each participant underwent genome-wide genotyping, with detailed procedures for genotyping, quality control, and imputation, all previously described elsewhere[12,13].

Similar to the CUIMC and MGBB biobanks, for every individual, ancestry was defined using a random forest-based machine learning approach, which assigned each sample to a continental ancestry group. We trained and tested the random forest model using subjects from the 1000 Genomes Project (1KG) with known ancestry labels[14]. The model utilized 10 PCs as the labeled feature matrix. We then applied the trained model to predict genetic ancestry for all datasets[12].

To define CKD case and controls in these three biobanks, we applied our validated CKD e-phenotyping algorithm[9]. Cases were defined as individuals with an eGFR < 60 ml/min/1.73 m² (using the 2021 CKD-EPI equation[15]) or those undergoing kidney replacement therapy (dialysis or kidney transplant). Controls had an eGFR ≥90 ml/min/1.73 m² and no evidence of CKD based on diagnostic or procedure codes. To avoid case-control misclassification due to age-related eGFR decline and limited ability to perform EHR data mining in these biobanks as compared to our discovery cohorts, we excluded individuals with eGFR between 60–90 ml/min/1.73 m² from the case-control cohorts.

To further classify individuals with CKD who were likely to have it secondary to FSGS/SRNS, we developed a selection pipeline based on relevant ICD9 codes (**eTable 2, eFigure 2**). As a result, we generated three cohorts in our replication datasets that aligned with the discovery cohorts: FSGS/SRNS, non-FSGS CKD, and Controls. Duplicate or related participants between the discovery dataset (CUIMC and MGBB) and eMERGE-III, were removed from the eMERGE-III cohort.

Statistical Analyses

In the discovery cohorts, pairwise comparisons of M1 variant prevalence across phenotypic categories (FSGS/SRNS, non-FSGS CKD, and controls) were performed using Fisher's exact test and and regression analyses were performed using Firth's bias-reduced logistic regression, which applies a penalized likelihood approach to mitigate small-sample bias and issues of separation[16,17].These were applied separately within each genotype group (*APOL1*-HR and *APOL1*-LR) for each phenotype, using the logistf function in R v4.5.1. All odds

© 2026 Martinelli E et al. *JAMA Network Open*.

ratios were derived from the same multivariable model and represent independent associations adjusted for all other covariates included in the model. Covariates included sex and genetic ancestry in the full-cohort models, since the M1 variant, which can be observed on both G2 and, at much lower frequency, on G0 haplotypes, displays different minor allele frequency across populations. Conversely, models restricted to individuals of African ancestry were adjusted for sex only. Genetic ancestry was modeled as a binary variable (African vs non-African).

In the external biobank datasets, M1 variant prevalence was compared across phenotypic groups using the Cochran-Mantel-Haenszel (CMH) test, with biobank (All of Us, eMERGE-III, and UK Biobank) as a stratifying variable. Pairwise comparisons were conducted between FSGS/SRNS vs controls, non-FSGS CKD vs controls, and FSGS/SRNS vs non-FSGS CKD using 2×2×3 contingency tables, using biobank and sex as stratifying variables.

Statistical significance was defined as a two-sided P- value of <0.05. All analyses (Fisher exact test, Firth's bias-reduced logistic regression, CMH) and forest plots were conducted with R 4.5.1 (fisher.test, logistf and mantelhaen.test functions) and the bar plots were generated using GraphPad Prism version 10.6.1 for Windows, (GraphPad Software, Boston, Massachusetts USA, www.graphpad.com).

Results conform to Strengthening the Reporting of Observational Studies in Epidemiology (STROBE) reporting guideline.

© 2026 Martinelli E et al. *JAMA Network Open*.

eAppendix

EHR review suggests non-*APOL1* causes for CKD in *APOL1*-HR M1 carriers

The large-scale human genetics studies reported above indicate that the presence of M1 protects against APOL1 kidney disease, especially FSGS/SRNS, by defining a G2-M1 non-risk haplotype. Consequently, individuals with CKD who have *APOL1*-HR and the M1 protective missense variant should have an alternative cause for their CKD.

To test this hypothesis, we conducted a detailed retrospective review of the EHR for all *APOL*1-HR cases with CKD who harbored the M1 variant in the CUIMC and MGBB biobanks. First, although the group of FSGS/SRNS was significantly depleted for M1, this variant was found in 2/320 (0.63%) cases. Detailed review of the EHR revealed presentations that would be unusual for FSGS/SRNS attributable to APOL1. Both cases had a pediatric onset of NS with one classified as congenital nephrotic syndrome and the other diagnosed at 8 years of age. The latter child showed no progression of kidney disease after at least 8 years of follow-up which is an atypical natural course for APOL1 FSGS/SRNS. Both early onset and non-progressive presentation suggest that the FSGS/SRNS phenotype in these two cases is driven by non-APOL1 mechanisms, and may simply represent the background prevalence of FSGS/SRNS in cases with *APOL1*-HR-M1.

In the non-FSGS CKD group with *APOL1*-HR-M1 genotypes, EHR mining identified an obvious alternative cause for CKD in nearly all cases (10/13, [77%], **eTable 12**). The diagnosis spanned from autoimmune to structural, to metabolic kidney diseases. Two kidney biopsies were available for in-depth pathology review. One case (#3), which was noted to have some FSGS features, had a membranoproliferative pattern attributable to hepatitis C viral infection (**eFigure 5A-D**); the other case (#9) showed mild deposition of renal amyloidosis (**eFigure5 E-H**). In both cases, the histological features of APOL1 kidney disease were absent. Among the cases that did not have an obvious non-APOL1 cause of CKD, two cases had a diagnosis of hypertensive kidney disease, of whom one case had non-nephrotic range proteinuria (case #7 and #12). A kidney biopsy was not performed in these two cases, preventing precise diagnosis. Finally, one case (#11) presented at age 74 with steroid sensitive nephrotic syndrome with perihilar FSGS and moderate thickening of the glomerular basement membrane (GBM), all not consistent with an *APOL1* kidney disease diagnosis.

© 2026 Martinelli E et al. *JAMA Network Open*.

In summary, among all the cases with *APOL1*-HR genotypes and M1, we were able to identify an alternative, non-*APOL1* mediated, cause of CKD or FSGS in nearly all individuals (12/15, 80%). Importantly, in the 3 cases in whom we could not identify an alternative cause of CKD, the diagnostic workup was incomplete.

© 2026 Martinelli E et al. *JAMA Network Open*.

eFigure 1. Design of the study

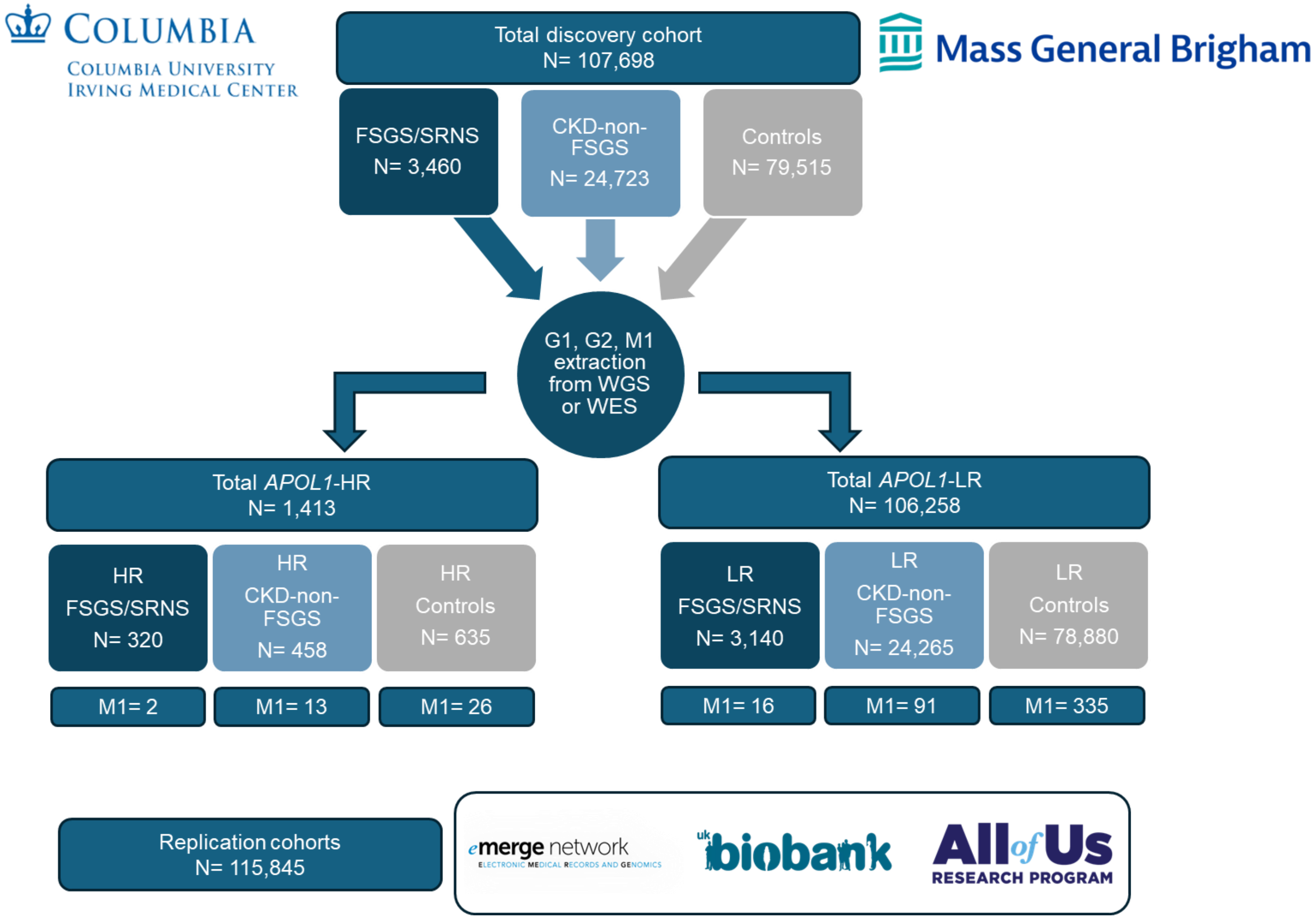

The overall study design was used to assess the protective effect of the *APOL1* M1 (p.N264K) variant in individuals at high risk (HR) for kidney disease due to the presence of *APOL1* risk alleles. The initial cohort included 107,698 individuals from Columbia University Irving Medical Center and Mass General Brigham Biobank, divided into three diagnostic categories: FSGS/SRNS, non-FSGS CKD, and controls. Whole-genome or whole-exome sequencing data were used to extract *APOL1* G1, G2, and M1 genotypes. Based on *APOL1* genotype, the cohort was stratified into HR and LR groups. Each was further subdivided into FSGS/SRNS cases, non-FSGS CKD cases, and controls. Additional external replication cohorts (eMERGE-III, UK Biobank, and All of Us) were included to validate findings. Abbreviations: HR: high-risk; LR: low-risk; FSGS: focal and segmental glomerulosclerosis; SRNS: steroid resistant nephrotic syndrome; CKD: chronic kidney disease.

© 2026 Martinelli E et al. *JAMA Network Open*.

eFigure 2. Case selection workflow for biobank replication analyses

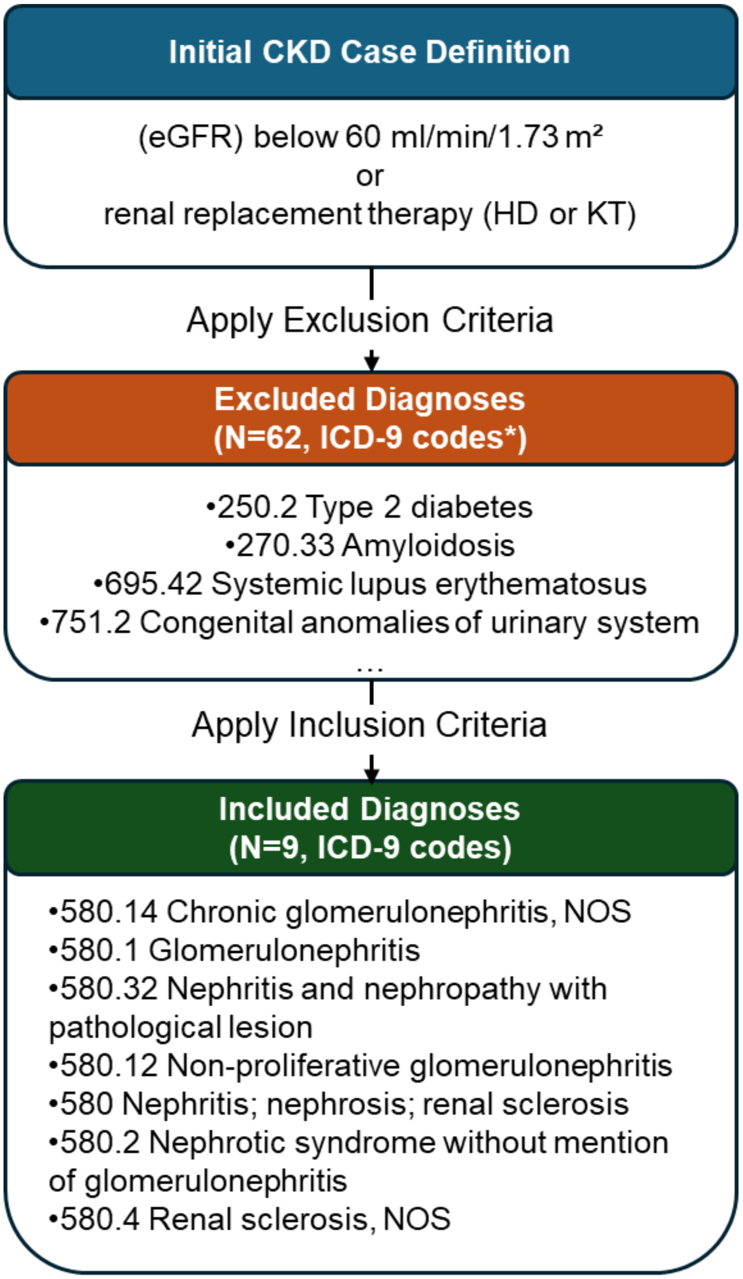

Flowchart used for the selection process of chronic kidney disease (CKD) cases in the biobank replication analysis. Participants were initially selected based on CKD status, defined by decreased eGFR or ongoing renal replacement therapy. Those with diagnoses indicating secondary causes of CKD (e.g., diabetic, autoimmune, or structural) were excluded based on a predefined list of 62 ICD-9 codes. The remaining participants were retained if they had at least one of the included ICD-9 codes associated with FSGS/SRNS. The complete list of excluded codes is provided in Table S2. Abbreviations: eGFR, estimated glomerular filtration rate; CKD, chronic kidney disease; HD, hemodialysis; KT, kidney transplant; NOS, not otherwise specified.

© 2026 Martinelli E et al. *JAMA Network Open*.

eFigure 3. Prevalence of M1 in APOL1 G2 containing HR genotypes.

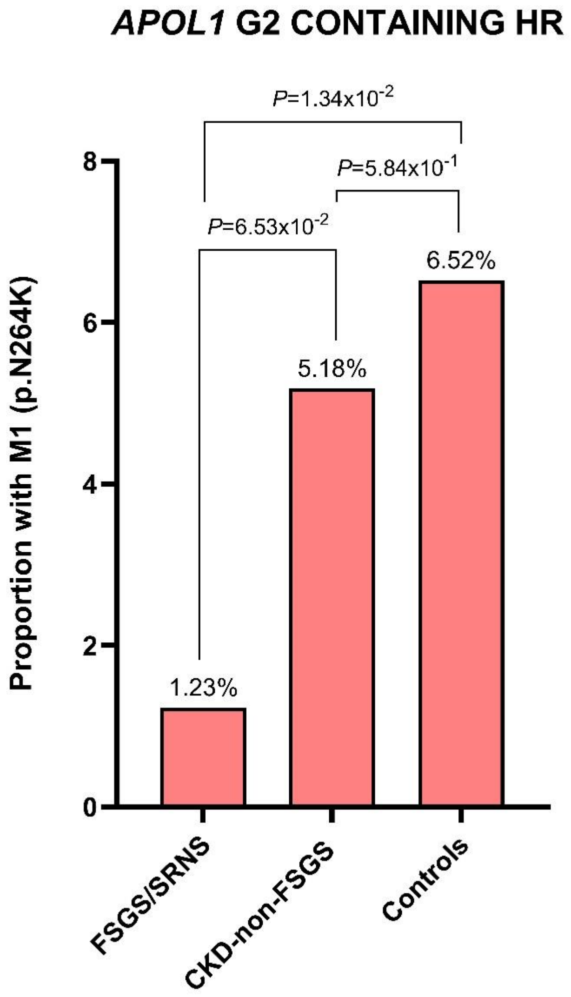

From the original analysis, we narrowed the cohort including only the G2 containing HR genotypes, replicating our findings. FSGS/SRNS vs. controls: 2/162 1.23% vs. 26/399 6.52%; Fisher's exact OR 0.18, 95%CI 0.02-0.74, P=8.8 x 10^{-3}, Firth regression OR 0.24, 95%CI 0.05-0.77 , P=1.34 x 10^{-2}, ; FSGS/SRNS vs non-FSGS CKD 2/162 vs 13/ 251 Fisher's exact OR 0.23, 95%CI 0.02-1.03, P=0.056; Firth regression OR 0.32, 95%CI 0.06-1.06, P=0.065; non-FSGS CKD vs controls 13/251 vs 26/ 403 Fisher's exact OR 0.8, 95%CI 0.38-1.68, P=0.61; Firth regression OR 0.83, 95%CI 0.41-1.61, P=0.58. Abbreviations: HR, high risk, WT, wild-type; FSGS, focal and segmental glomerulosclerosis; SRNS, steroid resistant nephrotic syndrome; CKD, chronic kidney disease.

© 2026 Martinelli E et al. *JAMA Network Open*.

eFigure 4. Prevalence of M1 across Biobanks and *APOL1* genotypes.

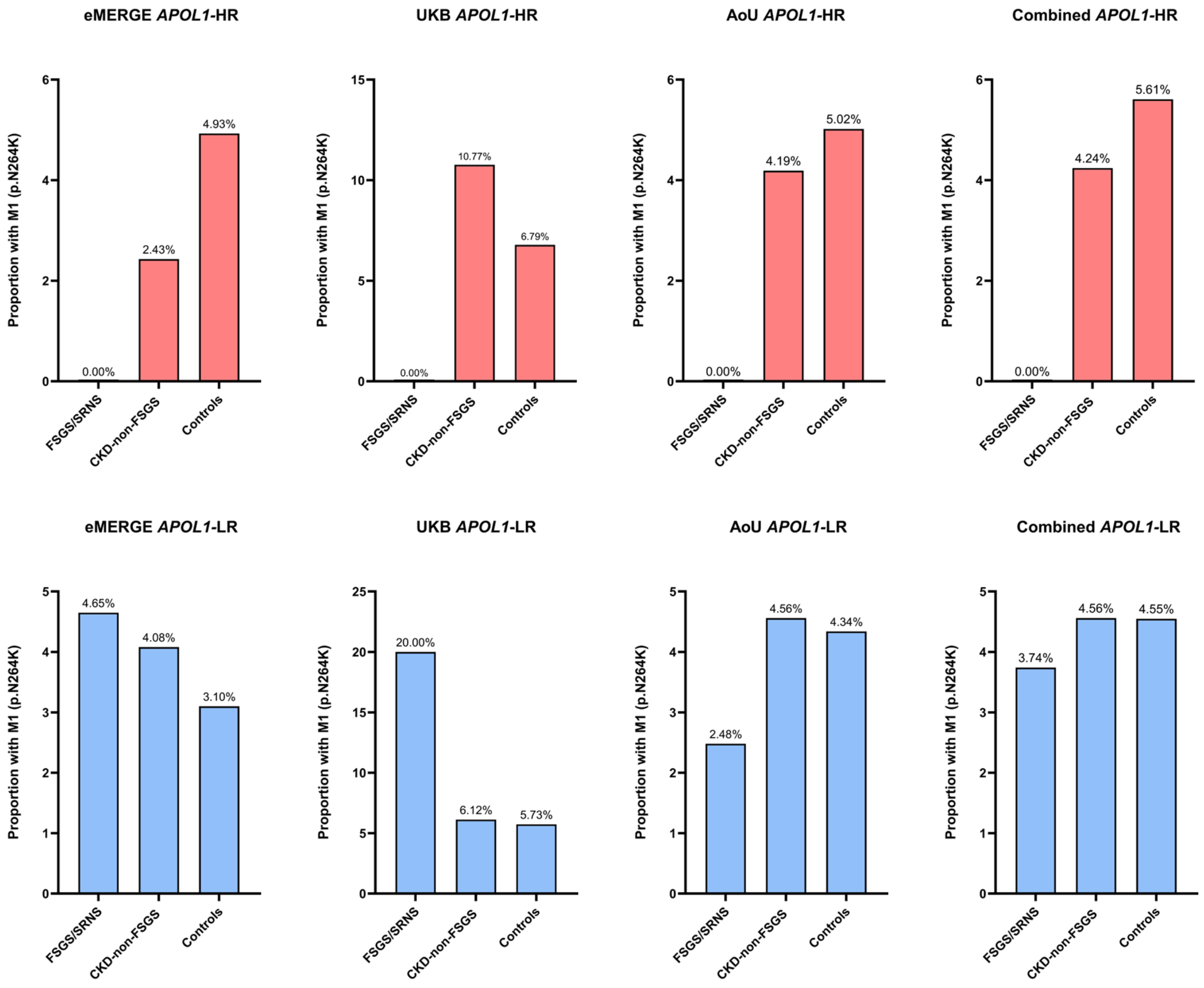

Bar plots show the proportion of individuals carrying the M1 variant (p.N264K) within APOL1 high-risk (HR, top row) and low-risk (LR, bottom row) genotypes across three large population cohorts (eMERGE-III, UK Biobank, All of Us) and the combined biobank dataset. Within each cohort, individuals were stratified by clinical diagnosis: FSGS/SRNS, CKD non-FSGS/SRNS, and unaffected controls. In all three cohorts, M1 prevalence was consistently absent among HR individuals with FSGS/SRNS and enriched in CKD and control groups. This pattern supports a protective association of M1 with FSGS/SRNS among HR individuals. In contrast, within the LR genotype, M1 was detectable across all diagnostic categories,

© 2026 Martinelli E et al. *JAMA Network Open*.

with no enrichment pattern in controls, not supporting a protective association. Abbreviations: HR, high risk, LR, low-risk; WT, wild-type; FSGS, focal and segmental glomerulosclerosis; SRNS, steroid resistant nephrotic syndrome; CKD, chronic kidney disease.

eFigure 5. Kidney biopsies review of case #3 and case #9

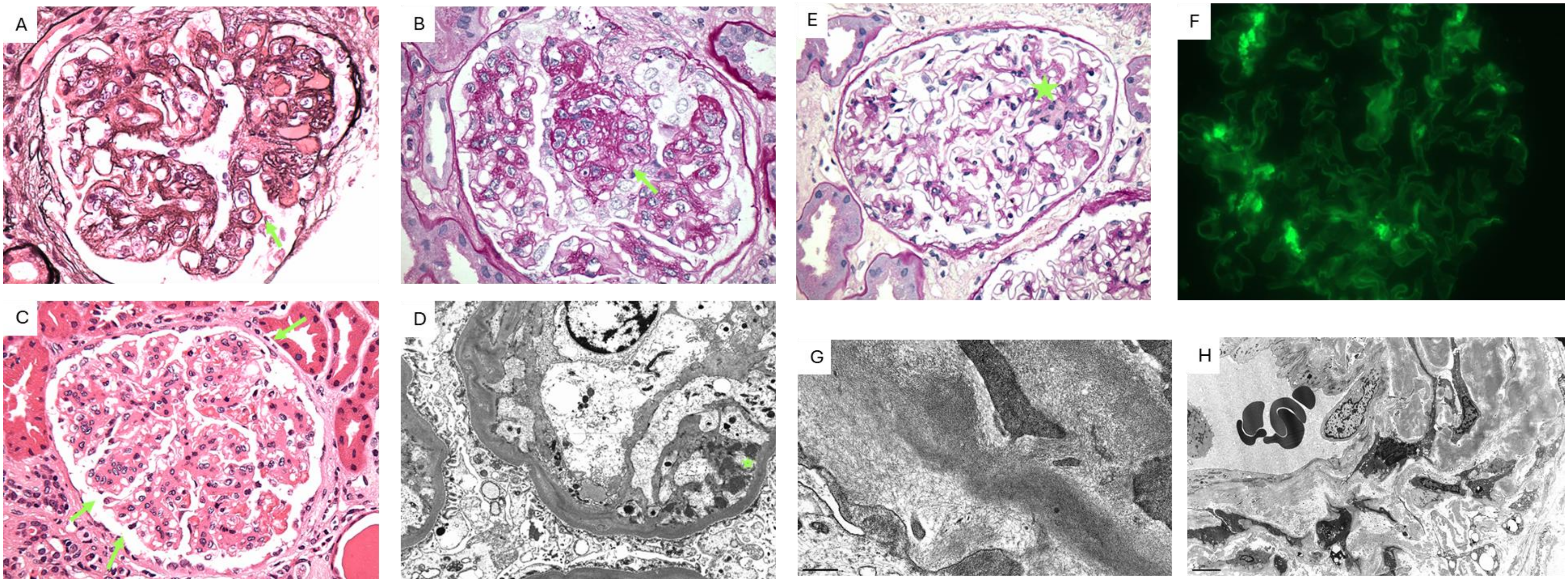

Histological review of case #3 revealed a membranoproliferative pattern in the context of HCV infection. A) Hematoxylin eosin staining showing increased mesangial hypercellularity (arrows), B) periodic acid–Schiff-stained slides showing mesangial expansion of matrix and cellularity (arrows). C) Jones methenamine silver staining highlighting duplications of the glomerular basement membranes (arrows). D) Electron microscopy showing disorganized mesangial deposits (star, original magnification, X5000).

Kidney biopsy review of case #9 showed amyloid deposition E) periodic acid–Schiff-stained slides pale mesangial deposition (star) (x400), F) Immunofluorescence positivity for IgG kappa. G) Electron microscopy showing fibrils deposition of 10 nm in the mesangial area (original magnification, X40000) H) Electron microscopy with lower power magnification x3000.

© 2026 Martinelli E et al. *JAMA Network Open*.

EXTENDED DATA TABLES

eTable 1. Exome capture kits used for Whole Exome Sequencing in the CUIMC cohort.

Exome capture kit	N
IDTERPv1,v2	20577
Roche	15368
Nextera	3132
Agilent	2316
Other	687

42,080 individuals from Columbia University Irving Medical Center (CUIMC) were subjected by whole exome sequencing (WES). In the table are listed the exome capture kits used for sequencing.

eTable 2. ICD9 codes

Refer to xls table

© 2026 Martinelli E et al. *JAMA Network Open*.

eTable 3. Allelic frequency M1 across ancestries and phenotypes

	AFR Alleles (AF)	AMR Alleles (AF)	EUR Alleles (AF)	EAS Alleles (AF)	SAS Alleles (AF)
Total M1	368 (2.1%)	94 (0.29%)	29 (0.02%)	0(0%)	0(0%)
FSGS/SRNS	12 (1.17%)	5 (0.26%)	1(0.03%)	0(0%)	0(0%)
Non-FSGS CKD	84 (2.13%)	13 (0.19%)	7(0.02%)	0(0%)	0(0%)
Control	272 (2.16%)	76 (0.32%)	21 (0.02%)	0(0%)	0(0%)
FSGS/SRNS LR	10 (2.10%)	5 (0.27%)	1 (0.03%)	0(0%)	0(0%)
Non-FSGS CKD LR	71 (2.25%)	13 (0.19%)	7 (0.02%)	0(0%)	0(0%)
Control LR	246 (2.17%)	76 (0.33%)	21 (0.02%)	0(0%)	0(0%)
FSGS/SRNS HR	2 (0.37%)	0(0%)	0(0%)	0(0%)	0(0%)
Non-FSGS CKD HR	13 (1.63%)	0(0%)	0(0%)	0(0%)	0(0%)
Control HR	26 (2.12%)	0(0%)	0(0%)	0(0%)	0(0%)

Abbreviations: AF; allelic frequency; AFR, African; AMR, Admixed American; EAS, East Asian, EUR, European, SAS, South Asian; CKD, chronic kidney disease; FSGS, focal and segmental glomerulosclerosis; SRNS, steroid resistant nephrotic syndrome; HR, high risk; LR, low risk.

© 2026 Martinelli E et al. *JAMA Network Open*.

eTable 4. Inverse association of M1 with FSGS/SRNS *APOL1*-HR genotypes, in both adjusted and unadjusted models.

Outcome	Variable	Adjusted		Unadjusted	
		OR	95%CI	OR	95%CI
FSGS/SRNS vs Controls	M1(p.N264K)	0.20**	0.04-0.63	0.15**	0.02-0.61
FSGS/SRNS vs Non-FSGS CKD	M1(p.N264K)	0.27*	0.05-0.90	0.21*	0.05-0.90
Non-FSGS CKD vs Controls	M1(p.N264K)	0.80	0.39-1.54	0.73	0.34-1.49

Results from adjusted (Firth's bias-reduced logistic regression) and unadjusted (Fisher exact test) analysis in *APOL1*-HR genotypes. Abbreviations: CKD, chronic kidney disease; FSGS, focal and segmental glomerulosclerosis; SRNS, steroid resistant nephrotic syndrome; OR, odds ratio; CI, confidence interval.
*P<0.05, **P<0.01

© 2026 Martinelli E et al. *JAMA Network Open*.

eTable5. Concordant M1 prevalence in *APOL1*-HR individuals with genetic African ancestry

APOL1-HR	M1	WT	%
FSGS/SRNS	2	271	0.73
Non-FSGS CKD	13	387	3.25
Controls	26	587	4.24

In the restricted analysis including individuals of genetic African ancestry only (N=8,779), we assessed the prevalence of M1 in APOL1-HR (N=1,286) individuals. M1 was significantly depleted in FSGS/SRNS cases compared to controls (Fisher's exact OR=0.17, 95%CI 0.02-0.67, P=5.6x10-3; Firth regression OR=0.20, 95%CI 0.04-0.63, *P*=3.70 x 10-3 with Firth). Cases with CKD non-FSGS were also depleted from M1 (Fisher's exact OR=0.76, 95%CI 0.35- 1.55, *P*=0.51; Firth regression OR= 0.79, 95%CI 0.39-1.54, P=0.50, with Firth) as compared to non-CKD controls but they had an intermediate prevalence of M1 and were 4-fold more likely to harbor M1 as compared to FSGS/SRNS cases (Fisher's exact OR=4.56, 95%CI 1.02-41.83, *P*=0.033;Firth regression OR=3.70, 95%CI 1.11-18.96 *P*=0.0315). Abbreviations: HR, high risk, WT, wild-type; FSGS, focal and segmental glomerulosclerosis; SRNS, steroid resistant nephrotic syndrome; CKD, chronic kidney disease.

© 2026 Martinelli E et al. *JAMA Network Open*.

eTable 6. Biobank-Based insights into M1 prevalence across *APOL1*-HR genotypes

Cohort	APOL1-HR	M1	WT	%
eMERGE-III				
	FSGS/SRNS	0	19	0
	Non-FSGS CKD	5	201	2.43
	Controls	10	193	4.93
UKB				
	FSGS/SRNS	0	3	0
	Non-FSGS CKD	7	58	10.77
	Controls	53	727	6.79
AoU				
	FSGS/SRNS	0	37	0
	Non-FSGS CKD	36	824	4.19
	Controls	68	1286	5.02
Combined				
	FSGS/SRNS	0	59	0
	Non-FSGS CKD	48	1083	4.24
	Controls	131	2206	5.61

Our replication cohort consisted in individuals of African genetic ancestry across the eMERGE-III, UKB and AoU biobank. Here we report the individual and aggregated results of M1 prevalence in HR cases (N=3,527). Pairwise comparisons of M1 prevalence using the Cochran-Mantel-Haenszel (CMH) test,stratified by biobanks and sex, showed no statistically significant differences. Specifically, there was no significant difference between FSGS/SRNS and controls (common OR= 0, *P*= 0.11), between non-FSGS CKD and controls (common OR= 0.89, 95% CI: 0.61–1.29, *P*= 0.58), or between FSGS/SRNS and non-FSGS CKD (common OR = 0, *P*= 0.25). Abbreviations: HR, high risk; WT, wild-type; FSGS, focal and segmental glomerulosclerosis; SRNS, steroid resistant nephrotic syndrome; CKD, chronic kidney disease.

© 2026 Martinelli E et al. *JAMA Network Open*.

eTable 7. M1 prevalence in *APOL1*-LR

APOL1-LR	M1	WT	%
FSGS/SRNS	16	3124	0.51
Non-FSGS CKD	91	23849	0.38
Controls	335	78868	0.42

Prevalence of M1 the *APOL1*-LR groups of FSGS/SRNS, non-FSGS CKD and controls. We did not find a statistically significant enrichment across the three phenotypes. FSGS/SRNS vs non-FSGS CKD Fisher's exact OR= 1.34, 95%CI 0.73 - 2.30, P= 0.29; Firth regression OR=1.26, 95%CI 0.71-2.10, P=0.40, FSGS/SRNS vs Controls Fisher's exact OR= 1.21, 95%CI 0.68-1.99, P= 0.48; Firth regression OR= 1.1, 95%CI 0.70- 1.94, P=0.47, non-FSGS CKD vs Controls Fisher's exact OR= 0.90, 95%CI 0.70-1.14, P= 0.389; Firth regression OR= 0.96, 95%CI 0.75-1.21, *P*=0.74). Abbreviations: LR, low-risk; WT, wild-type; FSGS, focal and segmental glomerulosclerosis; SRNS, steroid resistant nephrotic syndrome; CKD, chronic kidney disease.

© 2026 Martinelli E et al. *JAMA Network Open*.

eTable 8. Absence of association of M1 with FGSS/SRNS or Non-FSGS CKD in APOL1-LR genotypes

Outcome	Variable	Adjusted		Unadjusted	
		OR	95%CI	OR	95%CI
FSGS/SRNS vs Controls	M1(p.N264K)	1.21	0.70-1.94	1.21	0.68-1.99
FSGS/SRNS vs Non-FSGS CKD	M1(p.N264K)	1.26	0.71-2.10	1.34	0.73-2.30
Non-FSGS CKD vs Controls	M1(p.N264K)	0.96	0.75-1.21	0.90	0.70-1.14

Results from adjusted (Firth's bias-reduced logistic regression) and unadjusted (Fisher exact test) analysis in *APOL1*-LR genotypes. Abbreviations: CKD, chronic kidney disease; FSGS, focal and segmental glomerulosclerosis; SRNS, steroid resistant nephrotic syndrome; OR, odds ratio; CI, confidence interval.

© 2026 Martinelli E et al. *JAMA Network Open*.

eTable 9. M1 prevalence in *APOL1*-LR individuals of genetic African ancestry

APOL1-LR	M1	WT	%
FSGS/SRNS	10	228	4.20
Non-FSGS CKD	70	1505	3.17
Controls	239	5441	4.21

For APOL1-LR individuals of African ancestry (N=7,493), the prevalence of M1 was not different between FSGS/SRNS, CKD-non FSGS and controls. FSGS/SRNS vs non-FSGS CKD Fisher's exact OR=1.06, 95%CI 0.53-2.34, *P*= 1; FSGS/SRNS vs Controls Fisher's exact OR=1.00, 95%CI 0.52-2.15, *P*=1, non-FSGS CKD vs Controls Fisher's exact OR=94, 95%CI 0.79-1.40, *P*=0.67. Abbreviations: HR, high risk, WT, wild-type; FSGS, focal and segmental glomerulosclerosis; SRNS, steroid resistant nephrotic syndrome; CKD, chronic kidney disease.

© 2026 Martinelli E et al. *JAMA Network Open*.

eTable 10. *APOL1* G0G0 test for M1

APOL1-G0G0	M1	WT	%
FSGS/SRNS	10	2948	0.34
Non-FSGS CKD	51	22982	0.22
Controls	196	75778	0.26

Restricted analysis on M1 prevalence in G0G0 genotypes. The pairwise comparisons confirmed a non-independent protective role in this subset of cases. FSGS/SRNS vs non-FSGS CKD Fisher's exact OR=1.53, 95%CI 0.69--3.05, *P*= 0.223; Firth regression OR=1.40, 95%CI 0.67-2.66, *P*=0.36, FSGS/SRNS vs Controls Fisher's exact OR=1.31, 95%CI 0.62-2.47, *P*=0.36; Firth regression OR=1.19, 95%CI 0.59-2.14, *P*=0.59, non-FSGS CKD vs Controls Fisher's exact OR=0.95, 95%CI 0.62-1.17, *P*=0.37; Firth regression OR=0.94, 95%CI 0.68-1.28, *P*=0.71. Abbreviations: WT, wild-type; FSGS, focal and segmental glomerulosclerosis; SRNS, steroid resistant nephrotic syndrome; CKD, chronic kidney disease.

© 2026 Martinelli E et al. *JAMA Network Open*.

eTable 11. Biobank-Based insights into M1 prevalence across *APOL1*-LR genotypes

Cohort	APOL1-LR	M1	WT	%
eMERGE-III				
	FSGS/SRNS	2	41	4.65
	Non-FSGS CKD	28	658	4.08
	Controls	38	1186	3.10
UKB				
	FSGS/SRNS	2	8	20
	Non-FSGS CKD	12	184	6.12
	Controls	194	3192	5.73
AoU				
	FSGS/SRNS	4	157	2.48
	Non-FSGS CKD	203	4244	4.56
	Controls	446	9829	4.34
Combined				
	FSGS/SRNS	8	206	3.74
	Non-FSGS CKD	243	5086	4.56
	Controls	678	14207	4.55

M1 (p.N264K) was distributed overall similarly across *APOL1*-LR individuals belonging to eMERGE, UKB and AoU (N=20,428). Pairwise comparisons of M1 prevalence using the Cochran-Mantel-Haenszel (CMH) test, stratified by biobanks and sex, showed no statistically significant enrichment. Specifically, there was no significant difference between FSGS/SRNS and controls (common OR = 0.94, 95% CI: 0.46–1.92 P= 0.86), between non-FSGS CKD and controls (common OR = 1.08, 95% CI: 0.93–1.27, P= 0.33), or between FSGS/SRNS and non-FSGS CKD (common OR = 0.81, 95% CI: 0.39–1.66, P= 0.68). Abbreviations: LR, low-risk; WT, wild-type; FSGS, focal and segmental glomerulosclerosis; SRNS, steroid resistant nephrotic syndrome; CKD, chronic kidney disease.

eTable 12. Clinical characteristics of individuals with *APOL1*-HR genotypes carrying the M1 protective variant.

© 2026 Martinelli E et al. *JAMA Network Open*.

Case#	Gender	Age	Diagnosis
1	F	Adult	Early diabetic nephropathy, ANA positive
2	F	Pediatric	Non-nephrotic range proteinuria
3	F	Adult	MPGN, HCV-related
4	F	Adult	SLE – immune complex glomerulonephritis
5	M	Pediatric	CAKUT - obstructive uropathy
6	F	Pediatric	IgA nephropathy
7	M	Adult	Hypertensive Kidney disease without biopsy
8	M	Pediatric	CAKUT – posterior urethral valves
9	M	Adult	Renal amyloidosis
10	F	Adult	Pauci immune ANCA-negative glomerulonephritis
11	F	Adult	Steroid sensitive FSGS
12	M	Adult	Hypertensive Kidney disease without biopsy
13	F	Adult	Diabetic nephropathy

Cases are stratified by gender, age group (adult vs pediatric), and clinical diagnosis based on retrospective electronic health record review and, when available, histopathological findings. These findings underscore the importance of reassessing kidney disease diagnosis in APOL1-HR individuals who also carry the protective M1 variant and suggest that such individuals are more likely to have non-APOL1 kidney pathologies. Abbreviations: SLE, systemic lupus erythematosus; ANA, antinuclear antibody; CAKUT, congenital anomalies of the kidney and urinary tract; MPGN, membranoproliferative glomerulonephritis; HCV, hepatitis C virus; IgA, immunoglobulin A.

© 2026 Martinelli E et al. *JAMA Network Open*.

eReferences

1. Ren, Z. *et al.* ATAV: a comprehensive platform for population-scale genomic analyses. *BMC Bioinformatics* **22**, 149 (2021).
2. Koyama, S. *et al.* Decoding Genetics, Ancestry, and Geospatial Context for Precision Health. *medRxiv* (2023).
3. Milo Rasouly, H. *et al.* Exome analysis links kidney malformations to developmental disorders and reveals causal genes. *Nat Commun* **16**, 7290 (2025).
4. Martino, J. *et al.* Mouse and human studies support DSTYK loss of function as a low-penetrance and variable expressivity risk factor for congenital urinary tract anomalies. *Genet Med* **25**, 100983 (2023).
5. Wooden, B. *et al.* Natural History and Clinicopathological Associations of TRPC6-Associated Podocytopathy. *J Am Soc Nephrol* **36**, 274–289 (2025).
6. Manichaikul, A. *et al.* Robust relationship inference in genome-wide association studies. *Bioinformatics* **26**, 2867–73 (2010).
7. Chang, C.C. *et al.* Second-generation PLINK: rising to the challenge of larger and richer datasets. *Gigascience* **4**, 7 (2015).
8. Khan, A. *et al.* Medical Records-Based Genetic Studies of the Complement System. *J Am Soc Nephrol* **32**, 2031–2047 (2021).
9. Shang, N. *et al.* Medical records-based chronic kidney disease phenotype for clinical care and "big data" observational and genetic studies. *NPJ Digit Med* **4**, 70 (2021).
10. Bycroft, C. *et al.* The UK Biobank resource with deep phenotyping and genomic data. *Nature* **562**, 203–209 (2018).
11. Ramirez, A.H. *et al.* The All of Us Research Program: Data quality, utility, and diversity. *Patterns (N Y)* **3**, 100570 (2022).
12. Khan, A. *et al.* Polygenic risk alters the penetrance of monogenic kidney disease. *Nat Commun* **14**, 8318 (2023).
13. Khan, A. *et al.* Genome-wide polygenic score to predict chronic kidney disease across ancestries. *Nat Med* **28**, 1412–1420 (2022).
14. Genomes Project, C. *et al.* A global reference for human genetic variation. *Nature* **526**, 68–74 (2015).
15. Inker, L.A. *et al.* New Creatinine- and Cystatin C-Based Equations to Estimate GFR without Race. *N Engl J Med* **385**, 1737–1749 (2021).
16. Wang, X. Firth logistic regression for rare variant association tests. *Front Genet* **5**, 187 (2014).
17. Ma, C., Blackwell, T., Boehnke, M., Scott, L.J. & Go, T.D.i. Recommended joint and meta-analysis strategies for case-control association testing of single low-count variants. *Genet Epidemiol* **37**, 539–50 (2013).

© 2026 Martinelli E et al. *JAMA Network Open*.
