# Supplementary material for: Precision Diagnosis in APOL1 Kidney Disease With the p.N264K M1 Protective Variant
Source: JAMA Netw Open. 2026 Mar 11;9(3):e261452. doi: 10.1001/jamanetworkopen.2026.1452 (PMC12980251; doi:10.1001/jamanetworkopen.2026.1452)
Supplement: Supplement 3. — Nonauthor Collaborators [file jamanetwopen-e261452-s003.pdf]

Supplemental Online Content: Nonauthor Collaborators
*First name, last name, and suffix (if applicable) are required and will appear in PubMed.

*Group Name(s): Columbia Genomics Consortium							
***First Name and Middle Initial(s)**	***Last Name**	***Suffix (eg, Jr, III)**	Academic Degrees	Institution	Location (city, state/province, country)	Role or Contribution, eg, chair, principal investigator	Group (if more than 1 Group listed in the byline) and/or Subgroup (eg, Steering Committee)
Rando	Allikmets		MD, PhD	Department of Ophthalmology, Department of Pathology & Cell Biology, Columbia University	New York City, New York, USA.	genomic data	Columbia Genomics Consortium
Ansgar	Brambrink		MD, PhD	Department of Anesthesiology, Columbia University	New York City, New York, USA.	genomic data	Columbia Genomics Consortium
Matthew	Harms		MD	Department of Neurology, Columbia University College of Physicians and Surgeons	New York City, New York, USA.	genomic data	Columbia Genomics Consortium
Christine	Garcia		MD, PhD	Division of Pulmonary and Critical Care Medicine, Department of Internal Medicine, Columbia University	New York City, New York, USA.	genomic data	Columbia Genomics Consortium
Ali	Gharavi		MD	Department of Medicine, Vagelos College of Physicians & Surgeons, Columbia University	New York City, New York, USA.	genomic data	Columbia Genomics Consortium
David	Goldstein		PhD	Actio Biosciences Inc.	San Diego, California	genomic data	Columbia Genomics Consortium
Erin	Heinzen		PhD	Department of Genetics, School of Medicine; Division of Pharmacotherapy and Experimental Therapeutics, Eshelman School of Pharmacy, University of North Carolina	Chapel Hill, NC 27599, USA	genomic data	Columbia Genomics Consortium
Krzysztof	Kiryluk		MD	Department of Medicine, Vagelos College of Physicians & Surgeons, Columbia University	New York City, New York, USA.	genomic data	Columbia Genomics Consortium
Richard	Mayeux		MD, MSc	Department of Neurology, Columbia University Irving Medical Center, Gertrude H. Sergievsky Center, College of Physicians and Surgeons, Taub Institute for Research on Alzheimer's Disease and the Aging Brain, Columbia University Irving Medical Center, Columbia University	New York City, New York, USA.	genomic data	Columbia Genomics Consortium
Muredach	Reilly		MD	Division of Cardiology, Department of Medicine, Vagelos College of Physicians and Surgeons; The Irving Institute for Clinical and Translational Research; Columbia University	New York City, New York, USA.	genomic data	Columbia Genomics Consortium
Simone	Sanna-Cherchi		MD	Division of Nephrology, Department of Medicine, Columbia University Irving Medical Center	New York City, New York, USA.	genomic data	Columbia Genomics Consortium
Neil	Shneider		MD, PhD	Department of Neurology, Columbia University; Eleanor and Lou Gehrig ALS Center, Columbia University Medical Center	New York City, New York, USA.	genomic data	Columbia Genomics Consortium
H. Blair	Simpson		MD, PhD	Department of Psychiatry, Columbia University; New York State Psychiatric Institute	New York City, New York, USA.	genomic data	Columbia Genomics Consortium
Ronald	Wapner		MD	Division of Women's Genetics, Department of Obstetrics and Gynecology, Columbia University Irving Medical Center	New York City, New York, USA.	genomic data	Columbia Genomics Consortium
