# Supplementary material for: Precision Diagnosis in APOL1 Kidney Disease With the p.N264K M1 Protective Variant
Source: JAMA Netw Open. 2026 Mar 11;9(3):e261452. doi: 10.1001/jamanetworkopen.2026.1452 (PMC12980251; doi:10.1001/jamanetworkopen.2026.1452)
Supplement: Supplement 4. — Data Sharing Statement [file jamanetwopen-e261452-s004.pdf]

Data Sharing Statement

Martinelli. Precision Diagnosis in APOL1 Kidney Disease With the p.N264K M1 Protective Variant. *JAMA Netw Open*. Published March 11, 2026.
doi:10.1001/jamanetworkopen.2026.1452

Data
Data available: Yes
Data types: Deidentified participant data
How to access data: All data supporting the findings described in this manuscript are available in the article and in the Supplementary Information and from the corresponding author upon request. Genome sequencing data from the CureGN (Accession phs002480.v3.p3, https://www.ncbi.nlm.nih.gov/projects/gap/cgi-bin/study.cgi?study_id=phs002480.v3.p3), NEPTUNE(Accession: phs003210.v1.p1, https://www.ncbi.nlm.nih.gov/projects/gap/cgi-bin/study.cgi?study_id=phs003210.v1.p1), eMERGE network phase III (Accession: phs001584.v2.p2, https://www.ncbi.nlm.nih.gov/projects/gap/cgi-bin/study.cgi?study_id=phs001584.v2.p2) studies, and exome sequencing data from the Columbia University Genetic Studies of Chronic Kidney Disease (CKD) (Accession: phs001828.v1.p1, https://www.ncbi.nlm.nih.gov/projects/gap/cgi-bin/study.cgi?study_id=phs001828.v1.p1), is deposited in dbGaP. The UKBB genotype and phenotype data are available through the UKBB web portal at https://www.ukbiobank.ac.uk/. All researchers who wish to access the research resource must register with the UK Biobank. The AoU genotype, WGS, and phenotype data are available through the AoU researcher workbench at https://www.researchallofus.org/data-tools/workbench/. The researchers interested in accessing these data must complete registration with the AoU study. Both biobanks require institutional data use agreements as part of the registration process.
When available: With publication

Supporting Documents
Document types: None

Additional Information
Who can access the data: As detalied above
Types of analyses: As allowed by dbGaP and Biobanks above.
Mechanisms of data availability: dbgap requires application and approval. Individual biobank have their own regulations
